# Supplementary material for: An Evolutionary Perspective on Hox Binding Site Preferences in Two Different Tissues
Source: J Dev Biol. 2021 Dec 13;9(4):57. doi: 10.3390/jdb9040057 (PMC8705983; doi:10.3390/jdb9040057)
Supplement: Supplementary file 1 [file jdb-09-00057-s001.zip › jdb-1446669-supplementary.pdf]

Journal of *Developmental Biology*

MDPI

A

Short motif		longer motif		motif based on Selex
TTTA	TAAA	ATAAAT	ATTTAT	TGATTTAT
TTAA	AATT	ATAAAC	GTTTAT	TGATTAAT
TTGA	TCAA	AATTA	TAATT	TGATTGAT
TAAT	TAAT			TGATTTAC
				TGATAAAT

B

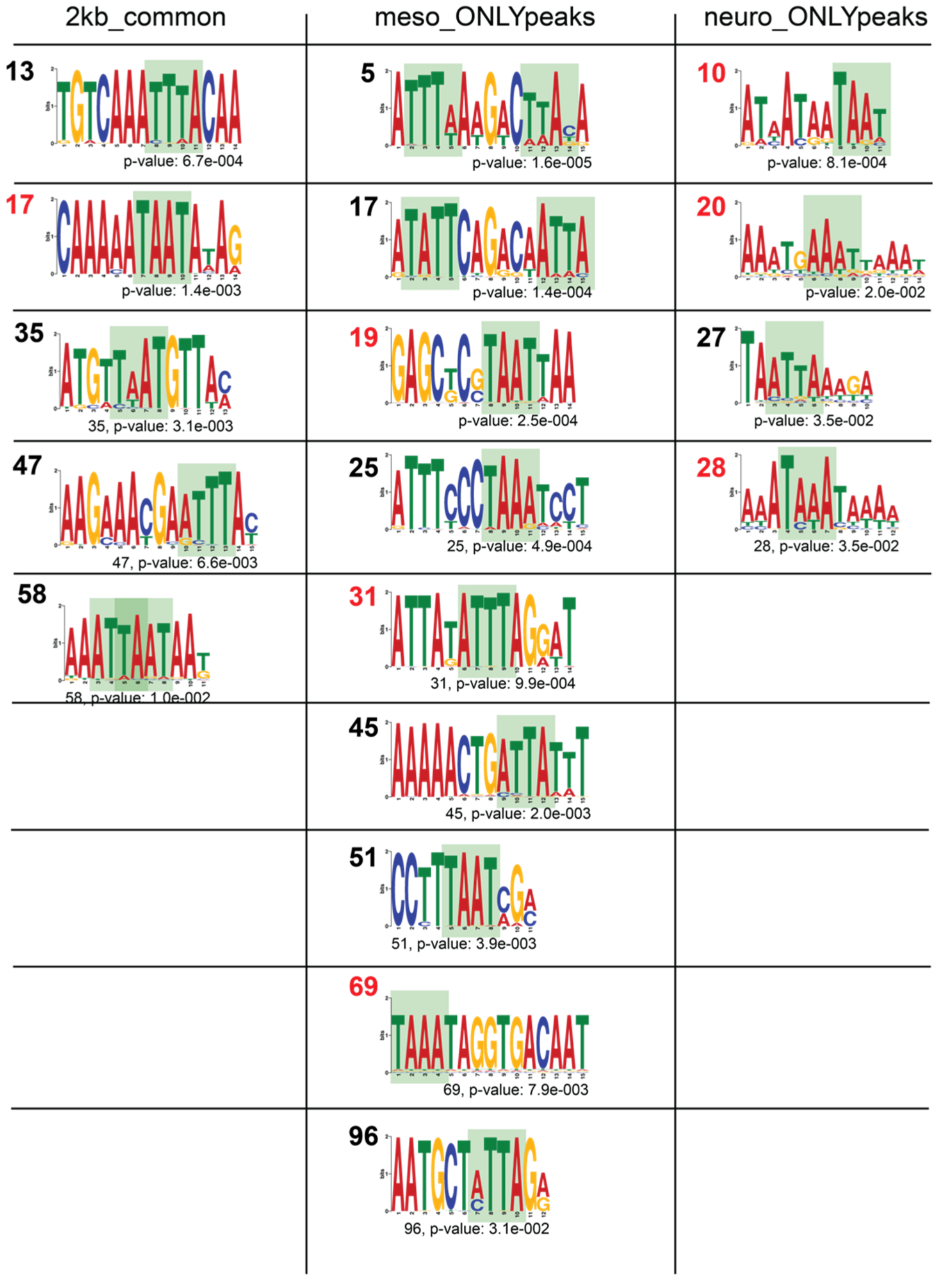

Figure S1: Novel motifs identified among different Ubx data sets. (**A**) Table of motifs that served as references to select novel Ubx binding motifs. (**B**) Novel Ubx binding motifs identified by the STREME sub-routine in the MEME suite using different data sets (2kb_common, meso_ONLYpeaks, neuro_ONLYpeaks) as input. Motifs with red labeled numbers were selected for closer analysis. Green boxes highlight the Ubx-like motif.

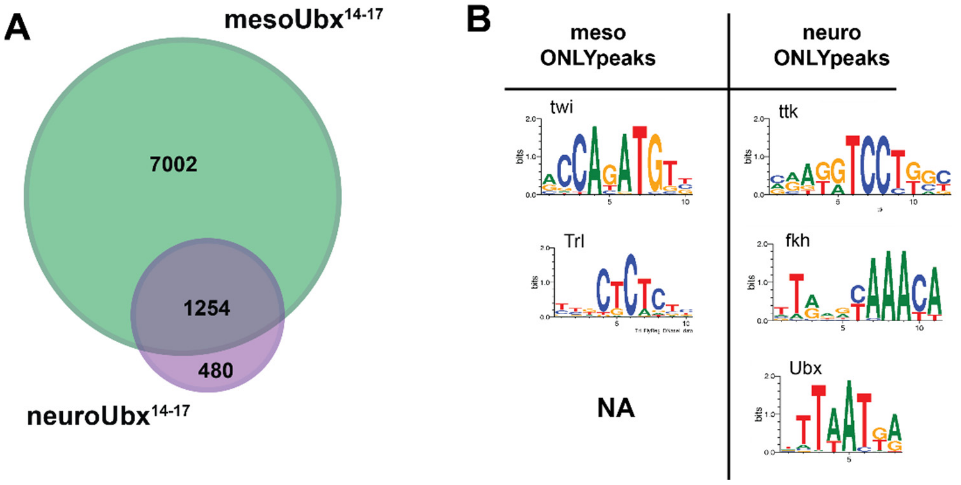

Figure S2. Analysis of the Ubx binding behavior during the differentiation stage (stage 14-17). (**A**) Venn diagram of Ubx peaks associated genes in the mesodermal and neuronal tissue, showing a substantial overlap of co-bound and potentially co-regulated genes (common genes). (**B**) Motif search based on an Analysis of Motif Enrichment (AME within the MEME suite) using the sequences from two categories meso_ONLYpeaks and neuro_ONLYpeaks derived from the common gene pool. The classical Ubx motif is not found in meso_ONLYpeaks. NA: not available.

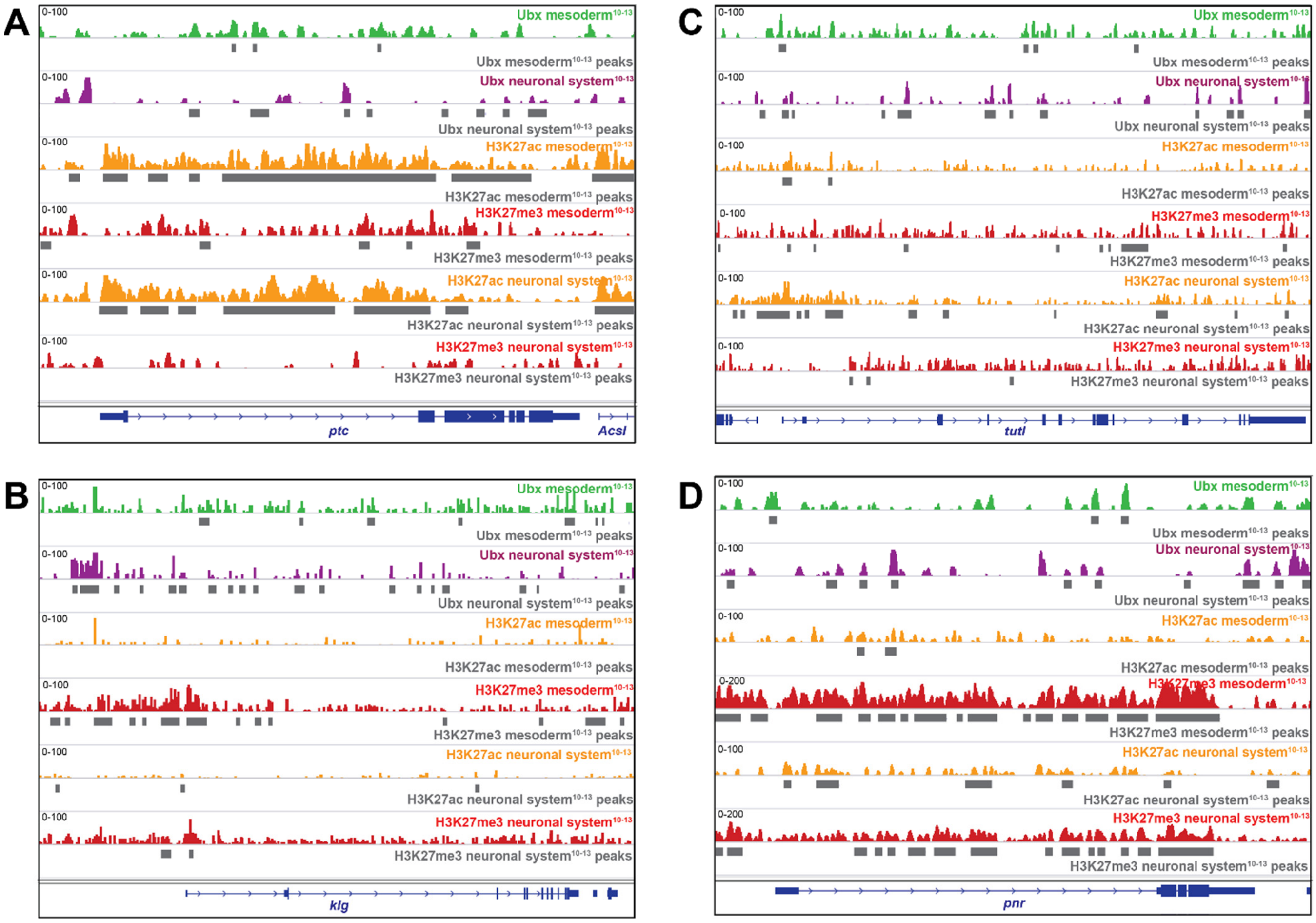

Figure S3. Genome view of selected examples to Figure 4. (**A**) Acetylation marks in the mesodermal and neuronal reads within the *patched* (*ptc*) locus. Scale: Ubx and histones 1-100. (**B**) Enriched methylation marks in mesodermal reads within the *klingon* (*klg*) locus. Scale: Ubx and histones 1-100. (**C**) Enriched acetylation marks in neuronal reads within the *turtle* (*tutl*) locus. Scale: Ubx and histones 1-100. (**D**) Methylation marks in neuronal and mesodermal reads withing the *pannier* (*pnr*) locus. Scale: Ubx 1:100, acetylations 1-100, methylations 1:200. Genome viewer annotations: Green: Ubx mesodermal reads stages 10-13, purple: Ubx neuronal reads stages 10-13, orange: H3K27ac mesodermal or neuronal reads stages 10-13, red: H3K27me3 mesodermal or neuronal reads stages 10-13, gray boxes: respected accepted peaks, blue: coding region.

Table S1. Characterization of the three different categories in Figure 2. Results were obtained from the DiffBind analysis.

	**mesoSpec**	**common genes**	**neuroSpec**
Category of genes (Enrichment according to WEADE)	Metabolism<br>Trafficking transport<br>Differentiation	Differentiation<br>Stimulus<br>Signalling pathways	Metabolism<br>Differentiation<br>Stimulus
Number of peaks	2589 (single: 931, multiple: 1676)	11772 (single: 1062, multiple: 10710)	4690 (single: 1936, multiple: 2754)
Location of peaks	Upstream: 42,31<br>Inside: 25,82<br>Downstream: 31,87	Upstream: 29,99<br>Inside: 49,23<br>Downstream: 20,78	Upstream: 25,53<br>Inside: 57,75<br>Downstream: 16,72
Biological GO-terms (top three hits)	Stress response to cadmium ion<br>Epithelial to mesenchymal transition<br>Negative regulation of membrane potential	Mesodermal fate commitment<br>Neuroblast development<br>Negative chemotaxis	Anion homeostasis<br>Basement membrane assembly<br>TOR signalling
molecular GO-terms (top three hits)	Juvenile hormone response element binding<br>Adenosine kinase activity<br>Oxidized DNA binding	Cell adhesion molecule binding<br>DNA-binding transcription activator activity<br>Actin binding	ABC-type xenobiotic transporter activity<br>Xenobiotic transmembrane transporter activity<br>Nucleoside binding
